# The predictive value of soluble osteoclast-associated receptor for the prognosis of acute coronary syndrome

**DOI:** 10.1038/s41598-021-91054-0

**Published:** 2021-06-01

**Authors:** Rong Wang, Jing Wang, Ling Xie, Hong-li Cai, Yi Zhang, Qing Zhang

**Affiliations:** 1 The Department of Nephrology, Affiliated Hospital 2 of Nantong University, Nantong, 226001 China; 2 The Department of Cardiology, Affiliated Hospital 2 of Nantong University, Nantong, 226001 China; 3 The Department of General Practice, Affiliated Hospital 2 of Nantong University, No. 6, Hai’er Xiang North Road, Chongchuan District, Nantong, 226001 China; 4 The Department of Scientific Research, Affiliated Hospital 2 of Nantong University, Nantong, 226001 China

**Keywords:** Cardiology, Diseases, Risk factors

## Abstract

At present, prognostic biomarkers of acute coronary syndrome (ACS) are fewer. The aim of this study was to explore the predictive value of soluble osteoclast-associated receptor (sOSCAR) level for the major adverse cardiovascular events (MACE) occurring within 30 days after ACS. From January to August 2020, a total of 108 patients with ACS who were admitted to our hospital, were enrolled in this study. Of the 108 patients, 79 were men and 29 women. Patient-related data, including age, sex, body mass index, history of type 2 diabetes, history of hyperlipidemia and serum sOSCAR level, were collected. All patients were followed up for 30 days. Based on MACE occurrence, the 108 patients were divided into MACE group (n = 17) and non-MACE group (n = 91). The baseline data were compared between the two groups, MACE-independent risk factors were identified by multivariate regression analysis, and the predictive value of sOSCAR for MACE occurring within 30 days after CAS was analyzed using receiver operating characteristic (ROC) curve. At the same time, according to the type of ACS, the 108 patients with ACS were divided into unstable angina (UA) group (n = 29), non ST-segment elevation myocardial infarction (USTEMI) group (n = 45) and ST-segment elevation myocardial infarction (STEMI) group (n = 34), and then the sOSCAR level and MACE incidence were observed in each group. The serum sOSCAR level was significantly lower in the MACE group [130(100,183)] than in the non-MACE group [301(220,370)] (*P* = 0.000). The area under ROC curve of sOSCAR level for MACE occurring within 30 days after CAS was 0.860 with 95%CI 0.782–0.919, *P* < 0.001. Multivariate regression analysis indicated that the sOSCAR level was an independent risk factor for the MACE occurring within 30 days after CAS (OR 0.26, 95%CI 0.087–0.777, *P* = 0.04). The MACE incidence (0%) was the lowest but the sOSCAR level was the highest in the UA group, while in the STEMI group, the MACE incidence (23.53%) was the higest but the sOSCAR level was the lowest among the UA, STEMI and NSTEMI groups. Serum sOSCAR level may be used as a predictor of MACE occurring within the short-term after ACS. The higher the sOSCAR level, the lower the MACE incidence.

## Introduction

Acute coronary syndrome (ACS) is characterized by rupture or invasion of coronary atherosclerotic plaques, thrombosis, myocardial ischemia, and it seriously threatens human life, health and safety^1^. At present, ACS-prognostic biomarkers are fewer because the ACS-pathological mechanisms is not clear. Osteoclast-associated receptor (OSCAR), a costimulatory receptor, is involved in osteoclast differentiation. OSCAR has two forms including soluble and membranous ones^2^. In the endothelial cells of vascular wall, there are OSCAR expression which is regulated by oxidized low density lipoprotein (oxLDL), and is involved in the occurrence and progression of atherosclerosis^3^. Therefore, we speculate that OSCAR plays an important role in the occurrence of cardiovascular disease. However, it is not clear whether soluble osteoclast-associated receptor (sOSCAR) level plays a predictive role in ACS prognosis. The aim of this study was to explore the predictive value of sOSCAR level for short-term prognosis of ACS.

## Subjects and methods

All study methods were approved by the Ethics Committee of the Second Affiliated Hospital of Nantong University (2019KS035) , and were performed in accordance with relevant guidelines and regulations. All the subjects enrolled into the study gave written informed consent to participate.

### Subjects and grouping

From January to August 2020, the patients who were admitted to our hospital for ACS, were collected. The inclusion criteria were the patients with ACS diagnosed according to clinical features, symptoms and coronary angiography^4,5^. The exclusion criteria were the patients with recent infections, osteoarthropathy, autoimmune diseases, malignant tumors, severe liver and kidney dysfunction, or chronic inflammatory diseases. Based on above inclusion and exclusion criteria, a total of 108 patients with ACS were enrolled in this study. Of the 108 patients, 79 were men and 29 women with a mean age of 66.94 ± 11.08 year. All patients were followed up for 30 days.

Based on the occurrence of major adverse cardiovascular events (MACE), the 108 patients were divided into MACE group (n = 17) and non-MACE group (n = 91).

According to the type of ACS, the 108 patients with ACS were divided into unstable angina (UA) group (n = 29), non ST-segment elevation myocardial infarction (USTEMI) group (n = 45) and ST-segment elevation myocardial infarction (STEMI) group (n = 34).

### Observation indices

The clinical data of all patients were recorded, including age, sex, history of smoking, history of hypertension, history of type 2 diabetes, history of hyperlipidemia, blood pressure and heart rate. In the morning of the next day after admission, fasting blood was taken from all patients for determining creatinine (Scr), troponin (TnI), total cholesterol (TC), high density lipoprotein (HDL), low density lipoprotein (LDL), triglyceride (TG), hypersensitive C-reactive protein (CRP), serum N-terminal pro-brain natriuretic peptide (NT-proBNP), and all patients receiced the examination of left ventricular ejection fraction (LVEF). Above clinical data first uderwent single factor analysis, and then the factors with significant differences between the two groups in the single factor analysis further underwent multivariate Logistic regression analysis.

The sOSCAR level and MACE incidence were observed in the UA, USTEMI and STEMI groups, respectively.

### Determination of sOSCAR level

In the morning of the next day after admission, 4 ml of fasting blood was taken from all patients for determining sOSCAR. The fasting blood was centrifuged at 2500r/min for 10 min, and then the supernatant was used to determine the sOSCAR level according to the instructions of enzyme linked immunosorbent assay kit provided by BioTSZ Company (Hong Kong, China).

### Follow-up

All patients were followed up by outpatient or telephone for 30 days after admission. The study endpoint was the occurrence of MACE. The MACE included death, heart failure, unstable angina and malignant arrhythmias. Within the 30 days, once the MACE occurred, the follow-up for the patient was stopped; otherwise the follow-up lasted 30 days.

### Statistical analysis

The measurement data with normal distribution were expressed as mean ± standard deviation (x ± s), and the measurement data with non-normal distribution were expressed as P50 (P25-P75). The enumeration data were expressed as percentage or frequency. Independent sample *t* test was used in the measurement data with normal distribution, *χ*^2^ test in the enumeration data, and Mann–Whitney U test in the measurement data with non-normal distribution. The receiver operating characteristic (ROC) curve was drawn, and the area under the curve (AUC) was used to evaluate the predictive value of sOSCAR for the MACE occurring within 30 days after ACS. Bivariate Logistic regression was used to identify independent risk factors of MACE in ACS. Statistical analysis was performed using SPSS 23.0 and Med-Calc (version 11.2.1; MedCalc) softwares. Statistical significance was established at *P* < 0.05.

## Results

### Comparisons of baseline data between the MACE group and non-MACE group

Age, Scr and NT-proBNP levels were significantly higher, but sOSCAR level and LVEF were significantly lower in the MACE group than in the non-MACE group (All *P* < 0.05) (Table 1).

**Table 1:** Comparisons of baseline data between the MACE group and non-MACE group [n, %, x ± s, P50 (P25-P75)].

	MACE group (n = 17)	Non-MACE group (n = 91)	*P* value
Age (years)	72.75 ± 10.15	63.72 ± 12.66	0.008
Gender (female)	7(41.18)	22(24.18)	0.147
Type 2 diabetes	3(17.65)	20(21.98)	0.487
Hypertension	13(76.47)	40(43.96)	0.116
Hyperlipoidemia	7(41.18)	35(38.46)	0.833
Smoking	5(29.41)	47(51.65)	0.150
Stroke	5(29.41)	13(14.29)	0.109
Heart rate (beats/min)	79.87 ± 15.96	79.96 ± 15.41	0.983
Systolic pressure (mmHg)	129.68 ± 20.73	135.74 ± 25.29	0.366
Diastolic pressure (mmHg)	77.87 ± 7.89	81.07 ± 18.96	0.254
Triglyceride (mmol/L)	1.62 ± 0.75	2.09 ± 0.86	0.311
Cholesterol (mmol/L)	3.90 ± 0.66	4.47 ± 1.19	0.075
HDL (mmol/L)	1.15 ± 0.31	1.15 ± 0.25	0.993
LDL (mmol/L)	2.30 ± 0.58	2.84 ± 1.10	0.065
sOSCAR (pg/ml)	130(100,183)	301(220,370)	0.000
Troponin I(µg/L)	2.53(0.72,7.17)	2.39(0.01,11.71)	0.625
hs-CRP	14.51(4.90,50.18)	7.75(2.35,21.33)	0.207
NT-proBNP ( pg/mL)	5314(2231,9334)	841(300,1715)	0.013
Creatinine (μmol/L)	87(70, 115)	72(61, 91)	0.032
LVEF (%)	49.73 ± 10.63	61.34 ± 8.01	0.001

*MACE* major adverse cardiovascular events; *LVEF* left ventricular ejection fraction; *NT-proBNP* N-terminal pro-brain natriuretic peptide; *hs-CRP* hypersensitivity C reactive protein; *sOSCAR* soluble osteoclast-associated receptor; HDL: high density lipoprotein; *LDL* low density lipoprotein.

### ROC curve of sOSCAR level for predicting MACE occurring within 30 days after ACS

**Figure 1:**
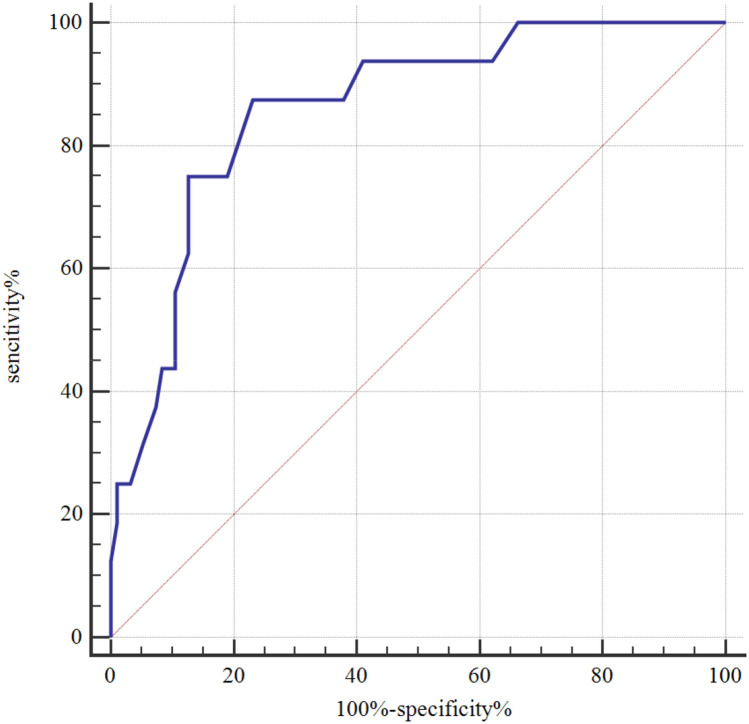
ROC curve of sOSCAR level for predicting MACE. Notes: *ROC* receiver operating characteristic; *sOSCAR* soluble osteoclast-associated receptor; *MACE* major adverse cardiovascular events.

The area under ROC curve of sOSCAR level for predicting MACE occurring within 30 days after CAS was 0.860 with 95%CI 0.782–0.919, *P* < 0.001, cut-off of 190 pg/ml, specificity of 76.84% and sensitivity of 87.50% (Fig. 1).

### Independent risk factors of MACE occurring within 30 days after ACS by multivariate Logistic regression analysis

The above 5 factors, including age, LVEF, sOSCAR, NT-proBNP and Scr, showed significant differences between the two groups, so the 5 factors, further underwent multivariate Logistic regression analysis. Multivariate Logistic regression analysis indicated that low serum sOSCAR (OR 0.26, 95% CI 0.087–0.777, *P* = 0.04), high NT-proBNP (OR 6.13, 95% CI 1.247–30.179, *P* = 0.01) and low LVEF value (OR 0.21, 95% CI 0.072–0.613, *P* = 0.03) were independent risk factors of MACE occurring within 30 days after ACS (Table 2).

**Table 2:** Independent risk factors of MACE occurring within 30 days after ACS by multivariate Logistic regression analysis.

Factors	OR values	95%CI	*P* values
NT-proBNP	6.13	1.247–30.179	0.01
LVEF	0.21	0.072–0.613	0.03
sOSCAR	0.26	0.087–0.777	0.04
Creatinine	2.13	0.319–14.153	0.25
Age	2.20	0.321–15.071	0.13

*MACE* major adverse cardiovascular events; *ACS* acute coronary syndrome; *NT-proBNP* N-terminal pro brain natriuretic peptide; *LVEF* left ventricular ejection fraction; *sOSCAR* soluble osteoclast-associated receptor.

### sOSCAR level in men and women

In this study, there were 79 men and 29 women. The sOSCAR level was 253.96 ± 131.01 pg/ml in men and 274.55 ± 101.08 pg/ml in women, and there was not significantly different between men and women in the sOSCAR level (*P* = 0.394).

### The sOSCAR level and MACE incidence in the UA, USTEMI and STEMI groups

According to the type of ACS, the 108 patients with ACS were divided into UA group (n = 29), USTEMI group (n = 45) and STEMI group (n = 34).

The sOSCAR level was 320 (245,390) pg/ml in the UA group, 270 (187,355) pg/ml in the USTEMI group and 250 (110,325) pg/ml in the STEMI group, respectively. The sOSCAR level was significantly higher in the UA group than in the STEMI group (*P* = 0.008), but it did not show significant differences between the UA group and USTEMI group (*P* = 0.279) as well as the STEMI group and USTEMI group (*P* = 0.332).

MACE occurred in 0 (0%, 0/29) patient of the UA group, in 9 patient (20%, 9/45) of the USTEMI group and in 8 patients (23.53%, 8/34) of the STEMI group.

## Discussion

ACS presents clinically as a group of clinical syndromes including the rupture or invasion of coronary atherosclerotic plaques and secondary complete or incomplete occlusive thrombosis. For the ACS, an acute and severe cardiovascular disease, the medical treatment is difficult, its mortality is high, and its clinical manifestation and prognosis are very various, so early judgement for ACS prognosis is very important for the patients with ACS. The human OSCAR, a cell surface molecule, is involved in osteoclast differentiation and bone metabolism^6^. OSCAR may play an important role in vascular inflammation or plaque vulnerability during atherosclerosis^7^. Our previous studies have also confirmed that sOSCAR has an independent correlation with the occurrence of ACS^8^. In this study, we further explored the relationship between sOSCAR level and ACS prognosis.

Our results indicated that the sOSCAR level was significantly lower in the MACE group than in the non-MACE group, and the area under ROC curve of sOSCAR level for predicting MACE occurring within 30 days after CAS was 0.860 with cut-off of 190 pg/ml, specificity of 76.84% and sensitivity of 87.50%, suggesting that MACE more easily occurs in the ACS patients with serum sOSCAR < 190 pg/ml. Multivariate Logistic regression analysis indicated that low serum sOSCAR level was an independent risk factor of MACE occurring within 30 days after ACS.

OSCAR is a member of the lymphocyte receptor complex family^3^, and is associated with osteoarticular diseases and osteoporosis^9,10^. Herman et al.^11^ found that the serum sOSCAR level was significantly lower in the patients with rheumatoid arthritis than in the normal individuals, and was negatively correlated with rheumatoid arthritis disease activity, so they believed that sOSCAR might have a protective effect. Goettsch et al.^12^ reported that OSCAR, an immune mediator and osteoclast differentiation regulator, is also involved in inflammatory reaction and cell activation during arteriosclerosis. Sinningen et al.^7^ have described that oxLDL, a atherosclerotic stimulator, can promote OSCAR expression on monocytes, so OSCAR may play an important role in vascular inflammation or plaque vulnerability during atherosclerosis. The oxLDL is from oxidation of low density lipoprotein (LDL), and is closely related to early atherosclerosis, because it can induce foam cell production, activate endothelial cell apoptosis and change gene expression, produce pro-inflammatory and atherosclerotic stimulation, and regulate costimulatory molecules in endothelial cells^13,14^. OSCAR is expressed in vascular endothelial cells and is regulated by oxLDL, oxLDL regulates OSCAR expression by calcium/nFAT dependent manner, OSCAR promotes the migration of monocytes across endothelial cells and participates in pro-inflammatory cascade reaction^3^. Therefore, OSCAR is involved in atherosclerotic diseases such as ACS probably with oxLDL as a mediator. Our previous studies have also confirmed that low sOSCAR is an independent risk factor of ACS^8^. This study further found that low sOSCAR level was closely related to MACE in ACS patients, so high serum sOSCAR level might have a protective effect. This is similar to the conclusion of Herman et al.^11^, but the specific protective mechanism remains to be further explored.

Based on our results, the MACE incidence (0%) was the lowest but the sOSCAR level was the highest in the UA group, while in the STEMI group, the MACE incidence (23.53%) was the higest but the sOSCAR level was the lowest among UA, NSTEMI and STEMI groups. Furthermore, the sOSCAR level was significantly higher in the UA group than in the STEMI group (*P* = 0.008). Therefore, we can conclude that the higher the sOSCAR level, the lower the MACE incidence.

There are some limitations in this study. Firstly, the sample size was small, and secondly the follow-up time was short. We will expand the sample size and prolong the follow-up time to further confirm the correlation between the serum sOSCAR level and MACE after ACS in future studies.

In summary, serum sOSCAR level is related to ACS prognosis and may be used as a biomarker to predict MACE occurrence after ACS. The higher the sOSCAR level, the lower the MACE incidence.
